# The effect of BMI on COVID-19 outcomes among older patients in South Korea: a nationwide retrospective cohort study*[Author-notes FN0001]

**DOI:** 10.1080/07853890.2021.1946587

**Published:** 2021-08-12

**Authors:** Yongtak Cho, Yongil Cho, Hyuk Joong Choi, Heekyung Lee, Tae Ho Lim, Hyunggoo Kang, Byuk Sung Ko, Jaehoon Oh

**Affiliations:** a Department of Emergency Medicine, College of Medicine, Hanyang University, Seoul, Republic of Korea; b Department of Emergency Medicine, College of Medicine, Yonsei University, Seoul, Republic of Korea

**Keywords:** COVID-19, body mass index, older patient, underweight

## Abstract

**Background: **The coronavirus disease 2019 (COVID-19) pandemic has caused deaths and shortages in medical resources worldwide, making the prediction of patient prognosis and the identification of risk factors very important. Increasing age is already known as one of the main risk factors for poor outcomes, but the effect of body mass index (BMI) on COVID-19 outcomes in older patients has not yet been investigated.

**Aim: **We aimed to determine the effect of BMI on the severity and mortality of COVID-19 among older patients in South Korea.

**Methods:** Data from 1272 COVID-19 patients (≥60 years old) were collected by the Korea Centers for Disease Control and Prevention. The odds ratios (ORs) of severe infection and death in the BMI groups were analyzed by logistic regression adjusted for covariates.

**Results:** The underweight group (BMI<18.5 kg/m^2^) had a higher OR for death (adjusted OR = 2.23, 95% confidence interval [95% CI] = 1.06–4.52) than the normal weight group (BMI, 18.5–22.9 kg/m^2^). Overweight (BMI, 23.0–24.9 kg/m^2^) was associated with lower risks of both severe infection (adjusted OR = 0.55, 95% CI = 0.31–0.94) and death (adjusted OR = 0.50, 95% CI = 0.27–0.91).

**Conclusions: **Underweight was associated with an increased risk of death, and overweight was related to lower risks of severe infection and death in older COVID-19 patients in Korea. However, this study was limited by the lack of availability of some information, including smoking status.

**KEY MESSAGES**
- Underweight is an independent risk factor of death in older COVID-19 patients.
- Overweight patients have a lower risk of death and severe infection than normal-weight patients.

## Introduction

First discovered in December 2019, severe acute respiratory syndrome coronavirus 2 (SARS-CoV-2) has caused a large number of casualties worldwide. As of February 2021, 108,246,992 patients had been diagnosed with coronavirus disease 2019 (COVID-19), and 2,386,717 had died worldwide [1]. Previous studies have suggested that risk factors such as older age, smoking, comorbidities, and obesity are associated with the severity of COVID-19 [2–5]. Among these risk factors, obesity is known not only for its risk in the context of COVID-19 but also for pulmonary infections, such as aspiration pneumonia and community-acquired pneumonia [6,7]. Numerous reports have defined the relationship between body mass index (BMI) and mortality due to various diseases. The results showed mostly J- or U-shaped relationships, indicating increased risks associated with both obesity and underweight [8–11]. Some studies identified reverse J-shaped relationships among older patients [12].

During this unprecedented crisis, many researchers have attempted to predict the risk of mortality due to COVID-19 according to BMI based on the background knowledge described above [13–17]. However, since the epicentres of the COVID-19 pandemic have mostly been in Western countries, the majority of results are from Europe or America. These results have clear limitations regarding their generalizability to Asians because the prevalence of obesity and distribution of BMI values differ substantially between Western and Asian populations. The World Health Organization (WHO) defines obesity as BMI ≥ 30 kg/m^2^ in Western populations and ≥25 kg/m^2^ in Asian populations because of the difference in body fat percentage. The criteria for underweight are the same in both populations [18,19]. Individuals with a BMI ≥ 25 kg/m^2^ in America account for approximately 68.8–74% of the total population, compared to 35% in South Korea. The percentage of the population classified as underweight is also different, accounting for 1.4–1.5% in America and 4.81% in South Korea [20–23]. This difference resulted in statistically nonsignificant findings of underweight status in previous studies of COVID-19 due to the small number of patients [13–17]. A large study from Mexico of 51,633 COVID-19 patients showed those with obesity had higher risks of hospitalization, intensive care unit (ICU) admission and intubation [24]. Another cohort study from the United Kingdom involving 20,133 patients also found obesity to be a risk factor of death [25]. Underweight patients were not investigated in either study. A recent cohort study conducted in South Korea and published in December 2020 emphasized the increased mortality of underweight patients [26]. However, the study was designed to include patients under the age of 20 years, and such patients cannot be classified according to adult BMI criteria. To our knowledge, there was only one death in the group of adult patients under age 50, which was too small of a sample size to be analyzed (Supplementary Table 1).

Because the rate of mortality due to COVID-19 in young patients is extremely low, and sporadic outbreaks have occurred in nursing homes in South Korea, we identified a need for further research focussed on older populations to help predict the prognosis of COVID-19. Herein, we conducted a nationwide cohort study to determine the effect of BMI on the severity and mortality of COVID-19 among older patients in Korea.

## Methods

### Data sources

Cohort data containing inpatient records of South Korean COVID-19 patients were released by the Central Disease Control Headquarters of the Korea Centres for Disease Control and Prevention. COVID-19 was defined by a positive result on a real-time reverse transcription-polymerase chain reaction (rRT-PCR) assay for SARS-CoV-2 with an upper respiratory tract specimen, with or without a positive result with a lower respiratory tract specimen. Data were collected by the patient information management office, the Korean Health Information Management Association and the National Medical Centre. Patients who were admitted to a hospital and released from quarantine between 20th January and 30th April 2020, were included. Patients were released from quarantine after complete recovery, which was defined by two consecutive negative PCR results with at least a 24-h interval between them. Data on age, sex, survival status, maximum disease severity, ICU admission, length of stay until release from quarantine or death, BMI, review of the system, initial vital signs, laboratory test results and comorbidities were obtained. Age was categorized into 10-year intervals. Maximum disease severity during admission was divided into 8 categories: no limit of activity, the limit of activity without oxygen, oxygen via nasal cannula, oxygen via a facial mask, non-invasive ventilation, invasive ventilation, multiorgan failure or extracorporeal membrane oxygenation (ECMO) and death.

### Study population

Data were collected from 5628 patients. Patients under 60 years of age and those with missing BMI data were excluded from the study because the mortality rate in patients under the age of 60 was extremely low (0.3%). Even though the WHO definition of older patients is 65 years or older, our data were provided in age groups with 10-year intervals, and to allow for convenient comparisons with previous studies, the cut-off of 60 years was deemed reasonable [17].

### Definitions of variables

BMI was calculated as the weight (kg) divided by the squared height (m^2^) and categorized according to the WHO Asian criteria into 5 categories: underweight (<18.5 kg/m^2^), normal weight (18.5–22.9 kg/m^2^), overweight (23.0–24.9 kg/m^2^), class I obesity (25.0–29.9 kg/m^2^), and class II obesity (≥30.0 kg/m^2^) [19].

### Outcomes

Our primary outcome was death before releasing from quarantine, and the secondary outcome was a severe infection. Severe infection was defined as the use of invasive mechanical ventilation or the development of multiorgan failure, the need for ECMO or death.

### Study design and statistical analysis

This is a retrospective cohort study using the data released by the Central Disease Control Headquarters of Korea Centres for Disease Control and Prevention. We described the characteristics of the given clinical factors and their association with BMI. Univariable analysis of the factors and outcomes was performed by Fisher’s exact test and the *χ*^2^ test for categorical variables. Covariates included factors that were statistically significant (*p*-value < .1) in the univariable analysis. Stratified analysis was used to identify confounders and effect modifiers. Multicollinearity was eliminated by using the generalized variance inflation factor. Multivariable logistic regression analysis was used and adjusted for the selected covariates. The normal weight group was set as a reference. Adjusted odds ratios (aORs) and 95% confidence intervals (CIs) were calculated, and a *p*-value < .05 was considered statistically significant.

Most of the previous studies have set the reference value as a BMI less than 25 kg/m^2^ due to the lack of cases in the underweight group and because the normal BMI standard of Westerners is 18.5–24.9 kg/m^2^. We designed an additional analysis utilizing the WHO standard criteria for BMI to make it easier to compare our results with those of existing studies. All statistical analyses were performed by R, version 4.0.2 (www.R-project.org).

### Ethical statement

The study was approved by the Hanyang University Institutional Review Board (HYUH 2020-07-037). The need to obtain informed consent was waived by the Institutional Review Board.

## Results

### Characteristics of the study population

**Figure 1.**
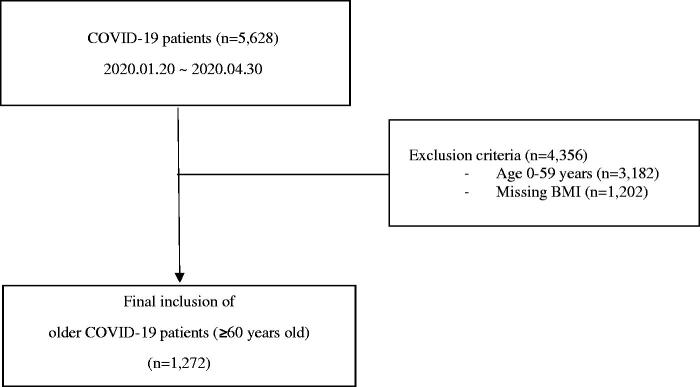
Flow chart of the study population. Definition of abbreviations: COVID-19: coronavirus disease 2019; BMI: body mass index

A total of 5628 COVID-19 patients were hospitalized and released from quarantine. A total of 3182 patients under the age of 60 and 1202 patients with missing BMI data were excluded. The final study population comprised 1,272 patients (≥60 years old) (Figure 1). The majority of the underweight (61.2%) and normal-weight patients (64.8%) were female, and the underweight group had the highest percentage of deaths and severe infections (22.7%). Cough was the most common symptom overall (43.7%). Hypertension was the most common comorbidity among older COVID-19 patients (44.8%). DM had the highest prevalence in the class I obesity group (29.8%), and dementia was most common in underweight patients (29.9%) (Table 1).

**Table 1.** Characteristics of study population by BMI group.

	Total (*n* = 1,272)	BMI (kg/m²)					*p*-Value
		<18.5 (*n* = 67)	18.5–22.9 (*n* = 488)	23.0–24.9 (*n* = 360)	25.0–29.9 (*n* = 326)	≥30 (*n* = 31)	
Age (years)							<.001
60–69	684 (53.8)	17 (25.4)	251 (51.4)	207 (57.5)	190 (58.3)	19 (61.3)	
70–79	378 (29.7)	28 (41.8)	136 (27.9)	111 (30.8)	95 (29.1)	8 (25.8)	
≥80	210 (16.5)	22 (32.8)	101 (20.7)	42 (11.7)	41 (12.6)	4 (12.9)	
Sex							
Female	750 (59.0)	41 (61.2)	316 (64.8)	198 (55.0)	171 (52.5)	24 (77.4)	.001
Male	522 (41.0)	26 (38.8)	172 (35.2)	162 (45.0)	155 (47.5)	7 (22.6)	
Outcome							
Severe infection^†^ (N/A = 9)	136 (10.7)	15 (22.7)	52 (10.7)	23 (6.4)	41 (12.7)	5 (16.1)	.001
Death	117 (9.2)	15 (22.7)	46 (9.5)	18 (5.0)	35 (10.9)	3 (9.7)	<.001
ICU (N/A = 8)	106 (8.3)	6 (9.0)	33 (6.8)	27 (7.5)	35 (10.9)	5 (16.1)	.137
Vital sign							
SBP (mmHg) (N/A = 1)							.001
<120	213 (16.7)	21 (31.3)	97 (19.9)	49 (13.6)	42 (12.9)	4 (12.9)	
120–139	429 (33.7)	14 (20.9)	166 (34.1)	127 (35.3)	107 (32.8)	15 (48.4)	
≥140	629 (49.4)	32 (47.8)	224 (46.0)	184 (51.1)	177 (54.3)	12 (38.7)	
HR (beats/min) (N/A = 2)		81.0 (73.5–90.0)	81.0 (71.0–92.0)	82.0 (74.0–95.0)	85.0 (76.0–95.0)	80.0 (73.0–88.0)	.004
Temp. (°C) (N/A = 3)		36.8 (36.5–37.3)	36.8 (36.5–37.4)	36.8 (36.5–37.2)	36.9 (36.5–37.3)	37.0 (36.7–37.3)	.262
ROS (N/A = 1)							
Fever	337 (26.5)	18 (26.9)	134 (27.5)	91 (25.3)	87 (26.7)	7 (22.6)	.944
Cough	556 (43.7)	32 (47.8)	201 (41.2)	160 (44.4)	147 (45.1)	16 (51.6)	.587
Sputum	415 (32.6)	24 (35.8)	156 (32.0)	118 (32.8)	107 (32.8)	10 (32.3)	.981
Sore throat	144 (11.3)	7 (10.4)	47 (9.6)	46 (12.8)	41 (12.6)	3 (9.7)	.593
Rhinorrhea	99 (7.8)	6 (9.0)	38 (7.8)	27 (7.5)	24 (7.4)	4 (12.9)	.847
Myalgia	191 (15.0)	4 (6.0)	80 (16.4)	55 (15.3)	48 (14.7)	4 (12.9)	.270
Fatigue	74 (5.8)	2 (3.0)	32 (6.6)	21 (5.8)	18 (5.5)	1 (3.2)	.754
Shortness of breath	251 (19.7)	13 (19.4)	88 (18.0)	64 (17.8)	78 (23.9)	8 (25.8)	.191
Headache	161 (12.7)	4 (6.0)	71 (14.5)	46 (12.8)	38 (11.7)	2 (6.5)	.225
Altered mentality	13 (1.0)	1 (1.5)	8 (1.6)	0 (0.0)	4 (1.2)	0 (0.0)	.188
Nausea/Vomiting	75 (5.9)	4 (6.0)	33 (6.8)	22 (6.1)	15 (4.6)	1 (3.2)	.722
Diarrhea	97 (7.6)	1 (1.5)	42 (8.6)	27 (7.5)	26 (8.0)	1 (3.2)	.271
Comorbidity							
DM	332 (26.1)	14 (20.9)	111 (22.7)	102 (28.3)	97 (29.8)	8 (25.8)	.137
HTN	570 (44.8)	23 (34.3)	178 (36.5)	167 (46.4)	183 (56.1)	19 (61.3)	<.001
Heart Dz (N/A = 4)	127 (10.0)	8 (11.9)	39 (8.0)	37 (10.3)	41 (12.6)	2 (6.5)	.260
Lung Dz	61 (4.8)	7 (10.4)	19 (3.9)	16 (4.4)	16 (4.9)	3 (9.7)	.122
CKD	26 (2.0)	1 (1.5)	11 (2.3)	5 (1.4)	7 (2.1)	2 (6.5)	.405
Cancer (N/A = 1)	53 (4.2)	3 (4.5)	27 (5.5)	9 (2.5)	11 (3.4)	3 (9.7)	.106
Liver Dz (N/A = 40)	27 (2.1)	3 (4.5)	8 (1.7)	5 (1.4)	10 (3.2)	1 (3.3)	.315
RDAD (N/A = 41)	12 (0.9)	0 (0.0)	9 (1.9)	2 (0.6)	1 (0.3)	0 (0.0)	.128
Dementia (N/A = 41)	117 (9.2)	20 (29.9)	62 (13.1)	20 (5.7)	15 (4.8)	0 (0.0)	<.001

All categorical variables are given in numbers (percentage). Percentages may not total to 100 due to rounding. Continuous nonparametric variables are given as the median (IQR). A Chi-square test or Fisher’s exact test was used for categorical variables. The Mann–Whitney *U* test was used for continuous nonparametric variables (*p*-value < .1 is statistically significant).

Severe infection^†^: outcome of invasive mechanical ventilation, extracorporeal membrane oxygenation, multiorgan failure or death.

Definition of abbreviations: BMI: body mass index; N/A: not available; ICU: intensive care unit; SBP: systolic blood pressure; HR: heart rate; Temp: temperature; ROS: review of system; DM: diabetes mellitus; HTN: hypertension; Dz: disease; CKD: chronic kidney disease; RDAD: rheumatic disease/autoimmune disease; IQR: interquartile range.

### Univariable analysis

The number of patients aged 80 years or older was the lowest (16.5%), but this group had the highest number of deaths (48.7%). The majority of the patients were in the normal weight group (38.4%), and the class II obesity group had the lowest percentage of patients (2.4%). We considered *p*-values <.1 statistically significant. Univariable analysis showed that sex, diabetes mellitus (DM), hypertension (HTN), heart disease, lung disease, chronic kidney disease (CKD), cancer, and dementia were associated with the outcomes (Table 2). Vital signs and symptoms were not included because they are considered mediator variables rather than covariates.

**Table 2.** Univariable analysis of severe infection^†^ and death.

	Total (*n* = 1272)	Severe infection (*n* = 136)	Nonsevere infection (*n* = 1127)	*p*-Value	Death (*n* = 117)	Survival (*n* = 1155)	*p*-Value
Age (years)				<.001			<.001
60–69	684 (53.8)	28 (20.6)	647 (57.4)		18 (15.4)	666 (57.7)	
70–79	378 (29.7)	49 (36.0)	329 (29.2)		42 (35.9)	336 (29.1)	
≥80	210 (16.5)	59 (43.4)	151 (13.4)		57 (48.7)	153 (13.2)	
Sex				<.001			.001
Female	750 (59.0)	56 (41.2)	691 (61.3)		51 (43.6)	699 (60.5)	
Male	522 (41.0)	80 (58.8)	436 (38.7)		66 (56.4)	456 (39.5)	
ICU (N/A = 8)	106 (8.3)	66 (48.5)	40 (3.5)	<.001	54 (46.2)	52 (4.5)	<.001
BMI (kg/m²)				.001			<.001
<18.5	67 (5.3)	15 (11.0)	51 (4.5)		15 (12.8)	15 (12.8)	
18.5–22.9	488 (38.4)	52 (38.2)	434 (38.5)		46 (39.3)	46 (39.3)	
23.0–24.9	360 (28.3)	23 (16.9)	335 (29.7)		18 (15.4)	342 (29.6)	
25.0–29.9	326 (25.6)	41 (30.1)	281 (24.9)		35 (29.9)	291 (25.2)	
≥30.0	31 (2.4)	5 (3.7)	26 (2.3)		3 (2.6)	28 (2.4)	
Comorbidity							
DM	332 (26.1)	58 (42.6)	272 (24.1)	<.001	52 (44.4)	280 (24.2)	<.001
HTN	570 (44.8)	85 (62.5)	483 (42.9)	<.001	73 (62.4)	497 (43.0)	<.001
Heart Dz (N/A = 4)	127 (10.0)	25 (18.4)	102 (9.1)	.001	21 (17.9)	106 (9.2)	.005
Lung Dz	61 (4.8)	13 (9.6)	48 (4.3)	.012	12 (10.3)	49 (4.2)	.007
CKD	26 (2.0)	11 (8.1)	15 (1.3)	<.001	9 (7.7)	17 (1.5)	<.001
Cancer (N/A = 1)	53 (4.2)	12 (8.8)	41 (3.6)	.009	12 (10.3)	41 (3.6)	.001
Liver Dz (N/A = 40)	27 (2.1)	3 (2.2)	24 (2.2)	1.000	3 (2.6)	24 (2.2)	1.000
RDAD (N/A = 41)	12 (0.9)	1 (0.7)	11 (1.0)	1.000	1 (0.9)	11 (1.0)	1.000
Dementia (N/A = 41)	117 (9.2)	34 (25.0)	83 (7.6)	<.001	34 (29.1)	83 (7.5)	<.001

All categorical variables are given in numbers (percentage). Percentages may not total to 100 due to rounding. A chi-square test or Fisher’s exact test was used for categorical variables. *p*-Value <.1 is statistically significant.

Severe infection^†^: outcome of invasive mechanical ventilation, extracorporeal membrane oxygenation, multiorgan failure or death.

Definition of abbreviations: BMI: body mass index; ICU: intensive care unit; N/A: not available; SBP: systolic blood pressure; HR: heart rate; Temp: temperature; ROS: review of system; DM: diabetes mellitus; HTN: hypertension; Dz: disease; CKD: chronic kidney disease; RDAD: rheumatic disease/autoimmune disease; IQR: interquartile range.

### Multivariable analysis

Multivariable logistic regression adjusted for the covariates is shown in Table 3. The underweight group had a 2.23-fold higher risk (95% CI, 1.06–4.52) of death than the normal weight group. The overweight group showed a lower risk of death, with an aOR of 0.50 (95% CI, 0.27–0.91), and classes I and II obesity were not significantly associated with the risk of death. The male population had a 2.28-fold higher mortality risk (95% CI, 1.49–3.52) than the female population. DM, HTN, CKD, cancer, and dementia were associated with a higher risk of death. Dementia had the highest aOR of 5.55 (95% CI, 3.28–9.33).

**Table 3.** Multivariable logistic regression results for risk of severe infection^†^ and death in COVID-19 patients aged 60 years or older.

	Severe infection^†^		Death	
	Adjusted OR	95% CI	Adjusted OR	95% CI
BMI (kg/m²)				
<18.5	1.99	0.96–3.96	2.23	1.06–4.52
18.5–22.9	Reference		Reference	
23.0–24.9	0.55	0.31–0.94	0.50	0.27–0.91
25.0–29.9	1.13	0.70–1.83	1.13	0.68–1.89
≥30.0	1.86	0.56–5.16	1.15	0.25–3.77
Sex				
Female	Reference		Reference	
Male	2.61	1.76–3.93	2.28	1.49–3.52
Comorbidity				
DM	1.72	1.15–2.58	1.94	1.26–2.98
HTN	1.94	1.29–2.94	1.96	1.27–3.06
Heart Dz (N/A = 4)	1.78	1.04–2.96	1.69	0.94–2.92
Lung Dz	1.91	0.89–3.86	2.13	0.96–4.41
CKD	4.45	1.73–11.17	3.55	1.30–9.20
Cancer (N/A = 1)	2.68	1.26–5.38	3.54	1.65–7.17
Dementia (N/A = 41)	4.55	2.72–7.55	5.55	3.28–9.33

Logistic regression was used to determine odds ratio. The 95% confidence interval was used to determine statistical significance.

Severe infection^†^: outcome of invasive mechanical ventilation, extracorporeal membrane oxygenation, multiorgan failure or death.

Definition of abbreviations: COVID-19: coronavirus disease 2019; OR: odds ratio; CI: confidence interval; BMI: body mass index; DM: diabetes mellitus; HTN: hypertension; Dz: disease; CKD: chronic kidney disease; N/A: not available.

With regard to severe infection, the underweight group and the normal weight group did not significantly differ. The overweight group had a lower risk of severe infection, with an aOR of 0.55 (95% CI, 0.31–0.94). Male sex, DM, HTN, heart disease, CKD, cancer, and dementia were associated with a higher risk of severe infection.

The results according to the WHO BMI criteria are given in Supplementary Table 2. The BMI < 18.5 kg/m^2^ group had a higher risk of death (aOR, 2.79; 95% CI, 1.35–5.54) and severe infection (aOR, 2.44; 95% CI, 1.19–4.76) than the BMI 18.5–24.9 kg/m^2^ group. Groups of 25.0–29.9 kg/m^2^ and ≥30 kg/m^2^ did not show any statistical significance.

## Discussion

Our results showed that older COVID-19 patients with a BMI under 18.5 kg/m^2^ had a higher risk of death than those in the normal BMI group. This result was not reported in most of the Western population-based studies, in which the numbers of underweight patients were small [13,17]. Our study had 67 underweight patients out of 1272 patients with confirmed cases of COVID-19 (5.3%), which was a higher proportion than those in most of the studies on BMI and COVID-19 published to date. A recent large cohort study of 6.9 million people in England in which 3% of the patients were underweight and 8.0% were of Asian ethnicity, a J-shaped association between BMI and death due to COVID-19 was identified, indicating an increased risk of death in people with BMI 20 kg/m^2^ or less, which supports our findings. However, the age-modified analysis showed an increased risk of death associated with increasing BMI, which could not show the relationships of the risk of death with underweight and overweight in older patients [27]. In our study, we identified BMI as an independent prognostic factor of death and severe infection in older COVID-19 patients.

Many previous studies have investigated the relationships between BMI and the incidence and mortality of various diseases [28–37]. According to a cohort study conducted in Denmark, the risk of infectious disease associated with BMI showed a U-shaped relationship, indicating a higher risk in underweight patients, especially for upper and lower respiratory infections [28]. The reasons underlying the increased risk associated with obesity have been reported, but the explanation for the increased risk associated with underweight is still under debate. While studies have shown that underweight increases all-cause mortality[10,30–32], some researchers have indicated that this is a bias caused by reverse causation [33–38]. However, several studies have shown that underweight is an independent risk factor after accounting for the potential for reverse causation [30,37–39]. There are several hypotheses regarding why underweight individuals have poor prognoses after contracting infectious diseases. For example, problems with the secretion of lymphocytes, albumin, and cytokines during periods of malnutrition and secretion of leptin in the context of sepsis [40–42]. By adjusting for factors that can be associated with reverse causation, such as sex and dementia, we found that underweight is independently associated with an increased risk of death, while overweight is actually associated with reduced risks of death and severe infection.

Although an attempt was made to include all possible confounders, information on smoking, ethnicity, concomitant infections and socioeconomic status was not available. Smoking is known to be a potential confounder of the risk of death in underweight patients [37,43]. However, some studies observed similar patterns regardless of smoking status [30,39,44]. A study by Williamson et al., which linked 17,278,392 adult patients from a registered health platform in England with 10,926 COVID-19-related deaths found a hazard ratio for mortality among current smokers of 0.89 (95% CI, 0.82–0.97), after full adjustment by other confounders. When adjusted for only demographic factors, a nonsignificant result was obtained, suggesting the effect of smoking on the outcomes of COVID-19 may not be as substantial as previously believed [45]. However, the lack of this information is a major limitation of our study. The results of some previous studies have suggested that non-white ethnicity is a risk factor for death in COVID-19 patients [45,46]. Ethnicity was missing from our data, which may have affected the validity of the results. However, the percentage of foreigners in Korea in 2019 was 3.4% according to Statics Korea, and the majority of them were Asian [47]. Unlike in other multiethnic countries, this confounder is unlikely to have caused bias. Socioeconomic variables (i.e. education level, occupation, residence) have also been considered as potential confounders in studies involving BMI [44,48]. However, in order to enable everyone to access all preventive measures regarding COVID-19, testing and treatment costs for patients in Korea have been covered by the National Health Insurance Service or government funding. Although the adjustment was incomplete, the provision of financial support means that these missing factors are relatively less critical.

There are several possible explanations for the results observed in older underweight and overweight patients in our study. The nutritional status of older patients in the underweight group was poor, and a protective role played by cholesterol in obese patients has been suggested. A previous meta-analysis showed that mortality in patients aged 60 years or older was reduced when their low-density lipoprotein level was high [49]. Another explanation of the increased mortality in underweight patients is the fat distribution. Obesity has been well studied as a major risk factor for cardiometabolic diseases. Interestingly, researchers have identified an even higher risk in some people with normal weight who have a higher visceral fat mass percentage and a lower gluteofemoral fat mass percentage. This suggests that not only the amount of fat mass but also its distribution can help predict the prognosis in patients with various diseases [50]. Considering the fact that the proportion of intraabdominal fat increases with age, the fat distribution in the older population could explain their poor prognosis [51]. The severity of COVID-19 and visceral obesity have been shown to be associated in several studies as well [52–54]. Because the findings have been based upon small numbers of patients, further research is needed to reveal whether the fat distribution is a better predictor of severe COVID-19 than overall obesity.

Our multivariable analysis did not show any significant associations of class I and II obesity with severe infection or death. This finding is contrary to the previous major studies. This is because only 3 deaths occurred in the BMI ≥30 kg/m^2^ group, and the released data categorized individuals with a BMI ≥30 kg/m^2^ into a single group. In previous studies in Western populations, the risk of mortality was the highest in the severely obese group (BMI ≥ 35 kg/m^2^), and severe obesity is not common in Korea [2,13,14,17,55]. However, research by Kim et al. with the same cohort data showed that BMI ≥25 kg/m^2^ was associated with a higher risk of death and high morbidity in patients aged 50 years or older. High morbidity was defined as the administration of oxygen via nasal cannula or facial mask, non-invasive ventilation, invasive ventilation, multiorgan failure/ECMO, and death. The result of overweight showed no significance, which is also different from the results of our study [26].

Lower risks of both death and severe infection in overweight patients are not novel findings. Previous studies regarding BMI in older individuals have shown that a higher BMI is associated with a reduction in mortality or morbidity. Despite the arguments about survivor bias and reverse causation affecting these findings, the protective effect of overweight in older adults is still a widely accepted concept [44,48,51]. Another study on BMI and all-cause mortality in Koreans showed that individuals with a BMI of 23.0–24.9 kg/m^2^ had the lowest risk of death. Among patients with respiratory diseases, a lower BMI was associated with a higher risk of death [30]. This phenomenon called the “obesity paradox”, is not fully understood [56]. Whether COVID-19 adheres to the “obesity paradox” has not yet been revealed, and there have been many reports that obesity increases the risk of death in COVID-19 patients. This finding may be due to the unique characteristics of the pandemic, including the shortages of staff and equipment [57]. Further studies are needed to clarify this matter.

This study has limitations. First, it is a retrospective study based on already collected data. Due to the nature of retrospective studies, it is impossible to completely control for missing data and confounding variables. A total of 1202 patients with missing BMI values were excluded from our study. The characteristics of the group of patients with missing data did not significantly differ from those of the study group, except for age, sex, DM, HTN and dementia (Supplementary Table 3). The patient information was collected from multiple centres, and the intention of this study was not to identify an association between BMI and COVID-19. Therefore, the missing data were omitted randomly, without any intentionality. Although it is not strongly suspected, the possibility of unchecked BMI at certain centres may have resulted in selection bias from data missing at random [58]. An overview of the missing data for potentially confounding variables has been provided above. Second, we were not able to accurately assess the patients who received invasive mechanical ventilation. Given that the variable was the maximum clinical severity, it is unknown whether the patients who died were on invasive mechanical ventilation. To correct this problem, a definition of severe infection was provided for this study. However, the inability to accurately identify the association between invasive mechanical ventilation and obesity, which has been found to be very strong, remains a limitation of this study [15,16]. Third, the numbers of cases and mortality due to COVID-19 in Korea are remarkably low compared to those in other countries. Although large-scale nationwide cohort data were used and statistically significant results were derived, the results had wide confidence intervals due to the low mortality rate. Fourth, this study only included hospitalized patients. However, unlike in many other countries, a shortage of beds was not a major issue in Korea, and most of the patients with confirmed cases of COVID-19 were referred to the hospitals for admission until the treatment centre for mild or asymptomatic patients opened on 2nd March 2020. This may have caused a potential bias due to the lack of data from the patients who were not hospitalized, although not great in number. Finally, this is a single-country study, so it may have limits in its application to Asians or individuals of other ethnicities.

In conclusion, underweight is a risk factor for death and overweight acts as a protective factor against death and severe infection in older Korean COVID-19 patients. The optimal BMI advised for older COVID-19 patients should be 23.0–24.9 kg/m^2^ to lower the risk of severe COVID-19 infection, and patients with BMI values under 18.5 kg/m^2^ should be observed and treated carefully.

## Supplementary Material

Supplemental Material

## Data Availability

Raw data were generated by the Korea Centre for Disease Control and Prevention. Derived data supporting the findings of this study are available from the corresponding author (JO) on request.
