# Supplementary material for: The effect of BMI on COVID-19 outcomes among older patients in South Korea: a nationwide retrospective cohort study*
Source: Ann Med. 2021 Aug 12;53(1):1293–302. doi: 10.1080/07853890.2021.1946587 (PMC8366651; doi:10.1080/07853890.2021.1946587)
Supplement: Supplemental Material [file IANN_A_1946587_SM4548.pdf]

Supplementary table 1. Age distribution of severe infection† and death

	Total (n=4,182)	Severe infection† (n=152)	Death (n=126)
Age (yr)			
20-29	956 (22.9)	0 (0.0)	0 (0.0)
30-39	478 (11.4)	1 (0.7)	0 (0.0)
40-49	598 (14.3)	1 (0.7)	1 (0.8)
50-59	878 (21.0)	14 (9.2)	8 (6.3)
60-69	684 (16.4)	28 (18.4)	18 (14.3)
70-79	378 (9.0)	49 (32.2)	42 (33.3)
≥80	210 (5.0)	59 (38.8)	57 (45.2)

All categorical variables are given in numbers (percentages). Percentages may not total to 100 due to rounding.

Severe infection† = outcome of invasive mechanical ventilation, extracorporeal membrane oxygenation, multiorgan failure or death

For Peer Review Only

Supplementary table 2. Multivariable logistic regression results for risk of severe infection† and death in COVID-19 patients aged 60 years or older (WHO BMI criteria)

	Severe infection†		Death	
	Adjusted OR	95% CI	Adjusted OR	95% CI
BMI (kg/m²)				
<18.5	2.44	1.19-4.76	2.79	1.35-5.54
18.5-24.9	Reference		Reference	
25.0-29.9	1.44	0.93-2.22	1.48	0.92-2.35
≥30.0	2.34	0.72-6.38	1.49	0.33-4.78
Sex				
Female	Reference		Reference	
Male	2.56	1.73-3.84	2.23	1.46-3.44
Comorbidity				
DM	1.67	1.11-2.49	1.87	1.21-2.86
HTN	1.88	1.26-2.84	1.89	1.22-2.94
Heart Dz (N/A=4)	1.77	1.04-2.94	1.68	0.94-2.89
Lung Dz	1.90	0.89-3.81	2.11	0.96-4.34
CKD	4.56	1.80-11.36	3.68	1.36-9.41
Cancer (N/A=1)	2.89	1.36-5.77	3.83	1.79-7.74
Dementia (N/A=41)	4.84	2.90-8.02	5.92	3.51-9.94

Logistic regression was used for odds ratio. The 95% confidence interval was used to determine statistical

For Peer Review Only

significance.

Severe infection† = outcome of invasive mechanical ventilation, extracorporeal membrane oxygenation, multiorgan failure or death

Definition of abbreviations: COVID-19 = coronavirus disease 2019; WHO = World Health Organization; BMI = body mass index; OR = odds ratio; CI = confidence interval; DM = diabetes mellitus; HTN = hypertension; Dz = disease; CKD = chronic kidney disease; N/A = not available

For Peer Review Only

Supplementary table 3. Characteristics of the patients with and without missing BMI data

		BMI data		
	Total	Missing	Available	p-value
	(n=5,628)	(n=1,202)	(n=4,426)	
Age (yr)				<0.001
<60	3,843 (68.3)	689 (57.3)	3,154 (71.3)	
≥60	1,785 (31.7)	513 (42.7)	1,272 (28.7)	
Sex				0.012
Female	3,308 (58.8)	745 (62.0)	2,563 (57.9)	
Male	2,320 (41.2)	457 (38.0)	1,863 (42.1)	
ICU (N/A=29)	189 (3.4)	43 (3.6)	146 (3.3)	0.719
Comorbidity				
DM (N/A=3)	691 (12.3)	185 (15.4)	506 (11.4)	<0.001
HTN (N/A=3)	1,201 (21.3)	337 (28.1)	864 (19.5)	<0.001
HF (N/A=3)	59 (1.0)	19 (1.6)	40 (0.9)	0.058
CCD (N/A=19)	179 (3.2)	45 (3.8)	134 (3.0)	0.248
Asthma (N/A=3)	128 (2.3)	28 (2.3)	100 (2.3)	0.962
COPD (N/A=3)	40 (0.7)	10 (0.8)	30 (0.7)	0.706
CKD (N/A=3)	55 (1.0)	11 (0.9)	44 (1.0)	0.941
Malignancy (N/A=4)	145 (2.6)	38 (3.2)	107 (2.4)	0.176
Liver Dz (N/A=326)	83 (1.5)	24 (2.0)	59 (1.4)	0.211

For Peer Review Only

RDAD (N/A=332)	38 (0.7)	7(0.6)	31 (0.8)	0.668
Dementia (N/A=329)	224 (4.0)	103 (8.6)	121 (3.0)	<0.001

All categorical variables are given in numbers (percentages). Percentages may not total to 100 due to rounding. A chi-square test or Fisher`s exact test was used for categorical variables (p-value < 0.05 is considered statistically significant).

Definition of abbreviations: BMI = body mass index; ICU = intensive care unit; N/A = not available; DM = diabetes mellitus; HTN = hypertension; HF = heart failure; CCD = chronic cardiac disease; COPD = chronic obstructive pulmonary disease; CKD = chronic kidney disease; Dz = disease; RDAD = rheumatic disease/autoimmune disease

For Peer Review Only
